# Microwave ablation triggers OX40L-mediated disruption of TNFRSF4+ Treg immunosuppressive activity

**DOI:** 10.3389/fimmu.2025.1637317

**Published:** 2025-10-23

**Authors:** Runqi Guo, Yufeng Wang, Jie Sun, Yuanming Li, Zhixin Bie, Xiaoguang Li

**Affiliations:** ^1^ Minimally Invasive Tumor Therapies Center, Beijing Hospital, National Center of Gerontology, Institute of Geriatric Medicine, Chinese Academy of Medical Sciences, Beijing, China; ^2^ Graduate School of Peking Union Medical College, Chinese Academy of Medical Sciences, Beijing, China

**Keywords:** microwave ablation, TNFRSF4+ Treg, tumor immunosuppression, tumor microenvironment, CD8+ T cells

## Abstract

**Objective:**

Regulatory T cells (Tregs) play a pivotal role in tumor immune evasion, and strategies to overcome their immunosuppressive activity are urgently needed. This study investigates the immunomodulatory effects of microwave ablation (MWA) on TNFRSF4+ Tregs, focusing on the OX40L/TNFRSF4 signaling axis as a potential therapeutic target.

**Methods:**

TNFRSF4+ Tregs were isolated from C57BL/6 mice and subjected to MWA-mimetic thermal stress. *In vitro* functional assays and *in vivo* LLC xenograft models were employed, with OX40 agonist intervention. Molecular mechanisms were analyzed via RT-qPCR, Western blot, and immunohistochemistry. The balance of tumor-infiltrating immune cells was quantified by multi-color flow cytometry.

**Results:**

MWA induced three key effects: (1) Phenotypic shift: decreased CTLA-4+ (P<0.0001) Treg subsets, but increased OX40L+ (P<0.01) in LLC cells; (2) Functional impairment: reduced Treg-mediated support for LLC proliferation, migration, and invasion; (3) Enhanced CD8+ T cell cytotoxicity. *In vivo*, MWA reshaped the tumor microenvironment by significantly increasing the intratumoral CD8+/Treg ratio (P<0.001), indicating a shift toward an anti-tumor inflammatory state. Mechanistically, MWA suppressed NF-κB/IκBα/TRAF6 signaling, and these effects were amplified by an OX40 agonist, suggesting the pathway is potentially OX40L-dependent.

**Conclusion:**

This study demonstrates that MWA disrupts Treg immunosuppression, likely by activating OX40L/TNFRSF4 signaling, and favorably alters the balance of effector to suppressor cells, providing a novel rationale for combining thermal ablation with OX40-targeted immunotherapies in cancer treatment.

## Introduction

Regulatory T cells (Tregs), characterized by CD4+CD25+FOXP3+ expression (1), are pivotal mediators of immune homeostasis that also drive tumor immune evasion through multiple mechanisms, including direct suppression of effector T cells, secretion of inhibitory cytokines (e.g., IL-10, TGF-β), and modulation of antigen-presenting cells (2, 3). In the tumor microenvironment, TNFRSF4 (OX40)+ Tregs exhibit enhanced immunosuppressive activity and correlate with poor prognosis across multiple cancers (4). The costimulatory molecule OX40 (CD134) and its ligand OX40L play context-dependent roles in immunity (5). In effector T cells, OX40 engagement promotes activation and survival, whereas in Tregs, it can destabilize FOXP3 expression and impair suppressive function (6, 7). Preclinical studies of OX40 agonists show potent antitumor effects, but clinical trials have yielded modest responses, possibly due to compensatory Treg activation (8, 9). This dichotomy highlights the need for strategies to selectively disrupt Treg OX40 signaling while sparing effector T cells.

Microwave ablation (MWA), a minimally invasive thermal therapy, achieves tumor control through coagulative necrosis induced by localized high temperatures (10). Emerging evidence suggests that MWA also modulates antitumor immunity, by releasing tumor antigens and danger-associated signals (11). Our previous work demonstrated that MWA enhances local T-cell abundance and alters monocyte interactions, thereby reshaping the immunosuppressive tumor microenvironment (TME) (12). However, its specific effects on immune cell subsets, particularly regulatory T cells (Tregs), key mediators of immune suppression in the TME, remain poorly understood (13). Although hyperthermia is known to alter lymphocyte function (14), the molecular mechanisms underlying temperature-dependent Treg regulation remain unexplored, leaving a critical gap in understanding how thermal ablation balances immune activation and suppression.

Despite the established roles of Tregs in tumor immunity and the emerging immunomodulatory effects of MWA, the precise impact of thermal ablation on Treg function remains unclear. Given the dual roles of OX40 signaling in both effector T cell activation and Treg suppression, we sought to investigate whether MWA could selectively target Treg immunosuppression through modulation of the OX40L/TNFRSF4 axis. This study was designed to elucidate the molecular mechanisms underlying thermal regulation of Treg function and explore potential synergies with OX40-targeted immunotherapy, offering new insights into combined physical and immunological approaches for cancer treatment.

## Materials and methods

### Experimental materials

EasySep™ Mouse CD8 Regulatory T Cell Positive Selection Kit (STEMCELL Technologies, #18782), True-Nuclear™ Mouse Treg Flow™ Kit (FOXP3 Alexa Fluor^®^ 488/CD4 APC/CD25 PE, BioLegend, #320029), PE anti-mouse CD152 (CTLA-4, BioLegend, #106305), APC anti-mouse CD134 (OX40, BioLegend, #119413), SuperScript III RT Reverse Transcription Kit (Invitrogen, #11752050), and SYBR Green qPCR Mix (Invitrogen, #4472920) were used in the study. The mouse Lewis lung carcinoma cells were purchased from Beyotime Biotechnology Company (#C7393). Efizonerimod alfa (EFA) was purchased from MedChemExpress Company(#HY-P99911). Female C57BL/6 mice (6–8 weeks old) were purchased from SPF Biotechnology (Beijing, China) and housed under pathogen-free conditions.

### Treg cell isolation and characterization

Fresh tumor tissues were rinsed with ice-cold PBS to remove blood and connective tissue. The tissue was minced into small fragments and centrifuged at 1,200 rpm for 5 min. The pellet was gently pressed through a 200-μm mesh sieve, washed with PBS, and filtered to obtain a single-cell suspension. The filtrate was centrifuged (1,200 rpm, 5 min), and the pellet was collected for further processing. CD4+CD25+FOXP3+ Treg cells were isolated using and True-Nuclear™ Mouse Treg Flow™ Kit according to the manufacturer’s protocol. CD8+ T cells were also sorted from splenocytes using the EasySep™ Mouse CD8a+ T Cell Isolation Kit. The purity of the isolated CD8+ T cell population was confirmed to be >95% by flow cytometry before use in cytotoxicity assays. Purity of CD4+CD25+FOXP3+ and TNFRSF4+ (CD134) Treg cells were confirmed by flow cytometry. All experiments were performed in triplicate.

### Cell culture and treatment

Treg cells and Lewis Lung Carcinoma (LLC) cells were maintained in complete RPMI-1640 medium supplemented with 10% fetal bovine serum (FBS), 2 mM L-glutamine, 1% penicillin-streptomycin, and 50 μM β-mercaptoethanol (for Tregs only). Cells were cultured at 37 °C in a humidified 5% CO_2_ incubator. Microwave Ablation (MWA) was performed using a clinically approved 2.45 GHz system with temperature feedback control. For *in vitro* applications, Treg cells in suspension were subjected to localized thermal stress at 37 °C, 45 °C, or 55 °C for 15 or 30 minutes in a preheated water bath with continuous agitation, followed by immediate transfer to 4 °C PBS to stabilize the heat-induced effects. The 37 °C condition served as the standard physiological baseline control, allowing for direct comparison to isolate the specific effects of hyperthermia. The system was calibrated to specific power outputs (e.g., 3W for 45 °C and 5W for 55 °C) to achieve and maintain stable target temperatures, as verified by infrared thermal imaging. Efizonerimod alfa was used at a concentration of 10 μg/mL for Treg cell treatment.

### Flow cytometry analysis

Cells were harvested and adjusted to 1×10^7^ cells/mL in PBS. For surface marker staining, 100 μL cell suspension was incubated with 1 μL fluorescent-conjugated antibody (PE anti-CTLA-4 or APC anti-OX40) for 30 min at room temperature in the dark. Cells were then washed twice with 2 mL PBS (1,000 rpm, 5 min each) to remove unbound antibodies. After final resuspension in 200 μL PBS, samples were analyzed using a BD FACSCanto II flow cytometer. Live cells were gated based on forward scatter (FSC) and side scatter (SSC) characteristics, followed by identification of CD4+CD25+FOXP3+ Treg populations. The frequency of target marker (TNFRSF4/OX40 or CTLA-4) expression was then quantified within this live Treg gate. In addition to Treg markers, tumor-infiltrating lymphocytes were also stained for CD8 to allow for the quantification of cytotoxic T cells, enabling the calculation of the CD8+/Treg ratio within the tumor. Appropriate controls including unstained cells and fluorescence-minus-one (FMO) samples were included for proper gating and compensation.

### CCK-8 assay

Cells were seeded in 96-well plates (5×10³ cells/well) and cultured for 24 h. After treatment, 10 μL CCK-8 reagent was added per well and incubated for 2 h at 37 °C. Absorbance was measured at 450 nm using a microplate reader. Blank control (medium only) and triplicate wells were included for normalization.

### Phase-contrast microscopy for cell morphology

LLC cells were seeded in 12-well plates (1×10^5^ cells/well) and allowed to adhere overnight. After treatment, cells were imaged at 100× magnification using an inverted phase-contrast microscope (Olympus IX73) equipped with a CCD camera. Five random fields per well were captured at 0, 6, 12, and 24 h post-treatment, maintaining consistent lighting and focus settings.

### Cell scratch wound healing assay

LLC cells were seeded in 12-well plates (5×10^5^ cells/well) and cultured to 90% confluence. A sterile 200 μL pipette tip was used to create a linear scratch, followed by PBS washing to remove debris. Cells were then cultured in serum-free medium with/without treatment. Scratch closure was monitored at 0, 12, and 24 h using phase-contrast microscopy (100×). Migration distance was quantified by measuring residual scratch width (ImageJ) and normalized to 0 h.

### Transwell invasion assay

LLC cells were seeded into Matrigel-coated (1:8 dilution) upper chambers (8 μm pores). Complete medium (10% FBS) was added to the lower chamber as chemoattractant. After 24 h at 37 °C, non-invaded cells were removed with cotton swabs. Invaded cells on the lower membrane surface were fixed (4% PFA), stained (0.1% crystal violet), and counted in 5 random fields (100×).

### Cytotoxicity assay

CD8+ T cells (effector) and LLC cells (target) were co-cultured at varying E:T ratios (0:1 to 16:1) in 96-well plates for 24 h. Cells were then fixed with 4% PFA and stained with 0.1% crystal violet. The remaining attached LLC cells were counted in three random fields (100×) per well.

### RT-qPCR

Total RNA was extracted using Trizol reagent, quantified by spectrophotometry (OD260/280 ≥ 1.8), and reverse-transcribed into cDNA (200 ng RNA) with oligo-dT/random hexamers (SuperScript III, 42 °C/60 min). RT-qPCR was performed in 20 μL reactions (SYBR Green Mix) with gene-specific primers under cycling conditions: 95 °C/5 min (initial denaturation), followed by 40 cycles of 95 °C/10 sec, 58 °C/20 sec, and 72 °C/20 sec. Melt curve analysis confirmed amplification specificity. Relative gene expression was calculated via 2^−ΔΔCt^ method using GAPDH as reference. All primers were synthesized by Sangon Biotech (Shanghai) and their sequences are listed in **Supplementary Table 1**.

### Western blot protocol

Cells were lysed in RIPA buffer, sonicated (3×10 sec pulses), and centrifuged (12,000 rpm, 15 min) to collect protein supernatants. Protein concentration was determined by BCA assay and adjusted to 5 mg/mL. Samples (20-50 μg) were separated by SDS-PAGE (10-12% gel, 80V for 2 h) and transferred to PVDF membranes (65V, 2 h). Membranes were blocked with 5% BSA for 1 h, incubated with primary antibodies (4 °C overnight) and HRP-conjugated secondary antibodies (1:3000, 1 h RT), then developed using ECL. Blots intensities were quantified by ImageJ. Detailed information on all antibodies used is provided in **Supplementary Table 2**.

### LLC xenograft tumor

C57BL/6 mice were subcutaneously injected with 1×10^6^ LLC cells bilaterally. When tumors reached ~50 mm³ (7 days post-inoculation), right-sided tumors underwent microwave ablation (55 °C for 15 min) with/without OX40 agonist treatment (10 mg/kg, i.p. every 3 days). Left-sided tumors served as internal controls. Tumor volumes (measured by caliper: 0.5×length×width²) and body weights were monitored every 3 days. Mice were euthanized at day 24 for tumor harvest and analysis. For *in vivo* tumor ablation, an MWA antenna was percutaneously inserted into murine LLC tumors under ultrasound guidance, delivering 5 W power for 2 minutes to achieve a 55 °C isotherm covering >90% of the tumor volume, as confirmed by real-time thermocouple monitoring. Animal experiments were performed with the approval of the Institutional Animal Ethics Committee and complied with the institution’s guidelines as well as the Regulations of the People’s Republic of China on the Administration of Laboratory Animals, ensuring humane care and use of laboratory animals.

### Immunohistochemistry

Tumor samples were fixed in 4% PFA, dehydrated through graded alcohols, and paraffin-embedded. Sections (4 μm) were deparaffinized (xylene) and rehydrated (graded ethanol→PBS). Antigen retrieval was performed in citrate buffer (pH 6.0) via microwave heating. After blocking endogenous peroxidase (3% H_2_O_2_) and nonspecific sites (5% serum), sections were incubated with primary antibodies (4 °C overnight), followed by HRP-conjugated secondary antibodies (37 °C, 30 min). Signal was developed using DAB (3–10 min), counterstained with hematoxylin, and mounted for imaging. Details of the primary and secondary antibodies used are listed in **Supplementary Table 2**.

### Statistical analysis

Quantitative data are presented as mean ± standard deviation (SD). Statistical analyses were performed using GraphPad Prism 9.0. For multiple group comparisons, one-way ANOVA followed by Tukey’s *post hoc* test was applied. A P-value <0.05 was considered statistically significant.

## Results

### Microwave ablation inhibited the viability of TNFRSF4+ Treg cells and its immunosuppressive function

**Figure 1:**
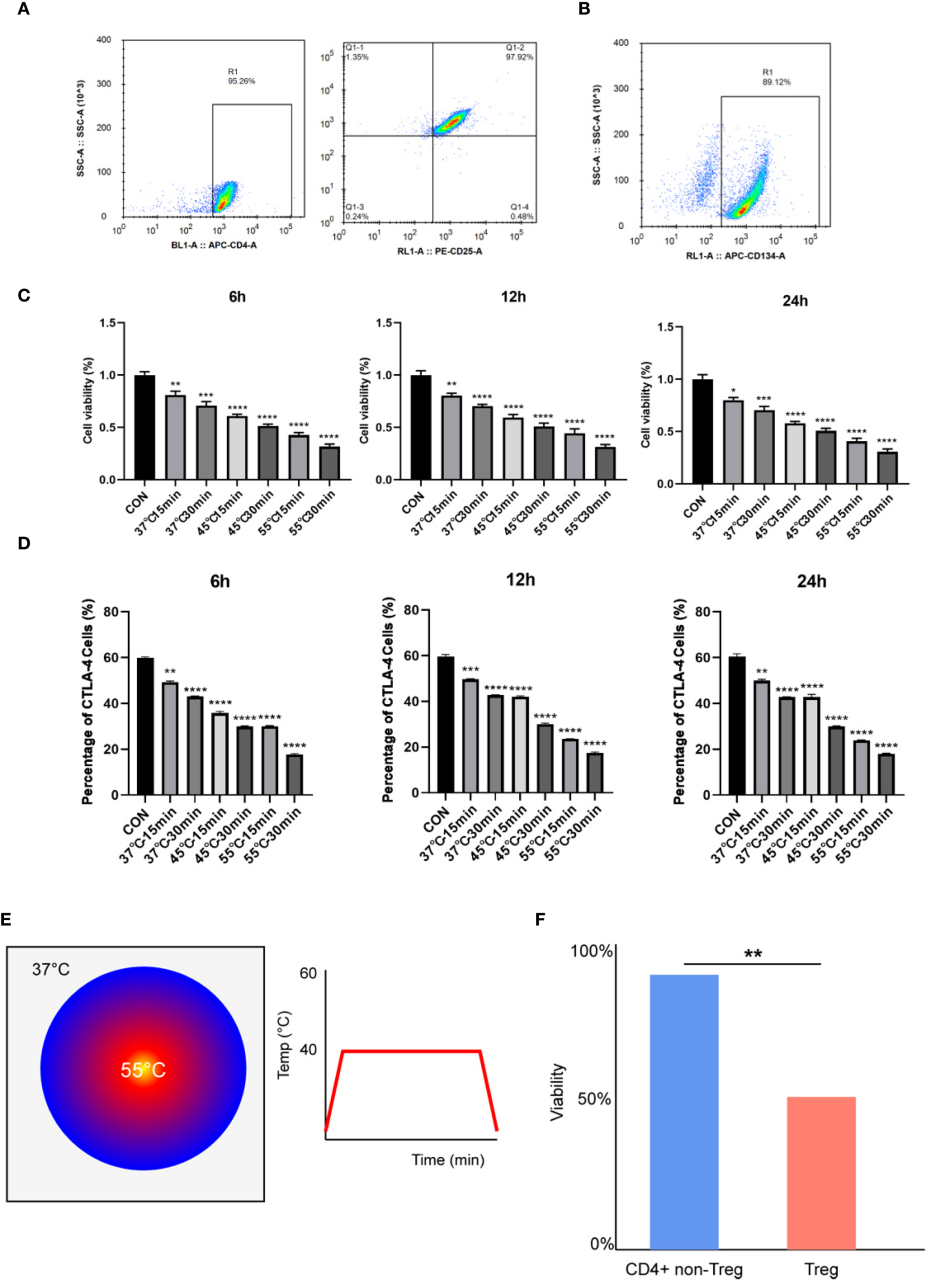
Microwave ablation selectively inhibited the viability of TNFRSF4+ Treg cells and its immunosuppressive function. **(A)** Treg cells (CD4+CD25+FOXP3+) were identified by flow cytometry, and the purity was more than 95%; **(B)** Flow cytometry revealed that about 90% of these Treg cells expressed TNFRSF4 (CD134); **(C)** CCK-8 assays demonstrated that MWA-mimetic heat stress significantly reduced Treg viability; **(D)** Heat stress significantly reduced CTLA-4+ Treg cell frequency (representative flow cytometry plots are shown in **Supplementary Figure 2**); **(E)** Representative infrared thermal image and time-temperature curve confirming stable and uniform heating during *in vitro* MWA. **(F)** Comparative viability of TNFRSF4+ Tregs and non-Treg CD4+ T cells after 15 min of heat stress, showing Tregs are more thermosensitive. *P<0.05, **P<0.01, ***P<0.001, ****P<0.0001, vs. CON group.

Treg cells (CD4+CD25+FOXP3+) were isolated from C57BL/6 mouse spleens using magnetic bead-based negative selection, achieving >95% purity (**Figure 1A**). Flow cytometry revealed that about 90% of these Treg cells expressed TNFRSF4 (CD134) (**Figure 1B**), confirming a highly enriched population for subsequent thermal stress experiments. To evaluate the impact of microwave ablation (MWA)-mimetic heat stress, TNFRSF4+ Treg cells were exposed to graded temperatures (37 °C, 45 °C, 55 °C) for 15 or 30 minutes. CCK-8 assays demonstrated that MWA-mimetic heat stress significantly reduced Treg viability in a temperature- and duration-dependent manner, with 55 °C/30 min causing the most severe reduction in viability (**Figure 1C**, P < 0.0001). Within each group, cell viability showed no significant differences between 6h, 12h, and 24h timepoints. Heat stress impaired Treg immunosuppressive function, as evidenced by downregulation of CTLA-4 (a critical inhibitory receptor). CTLA-4+ cell frequency decreased from 60% (CON) to 18% (55 °C 30 min) at 6h (**Figure 1D**, P < 0.0001). No recovery was observed over 24h, indicating sustained functional suppression. Infrared thermal imaging confirmed uniform and stable temperature delivery during the *in vitro* MWA treatment (**Figure 1E**). Notably, a comparative analysis revealed that TNFRSF4+ Tregs were significantly more sensitive to hyperthermia than the general non-Treg CD4+ T cell population (**Figure 1F**, P < 0.01), suggesting a degree of selective vulnerability.

### Heat-stressed TNFRSF4+ Treg cells exhibit diminished pro-tumorigenic functions

**Figure 2:**
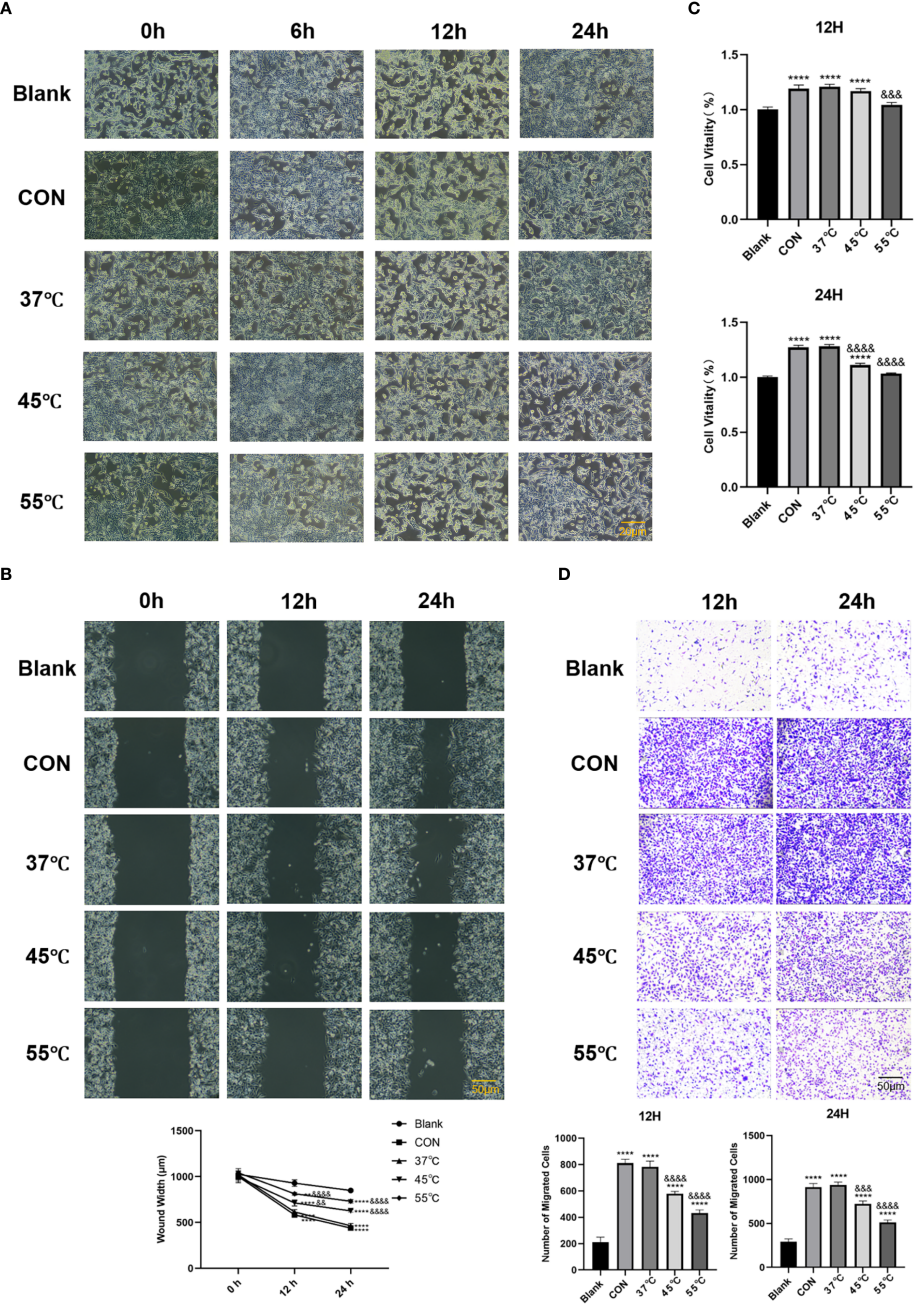
Heat-stressed TNFRSF4+ Treg cells exhibit diminished pro-tumorigenic functions. **(A)** High-resolution phase-contrast microscopy images showing morphological changes in LLC cells after co-culture with untreated or heat-stressed Treg cells (scale bar = 20 μm); **(B)** CCK-8 assay quantifying LLC cell proliferation at 12 and 24 hours post-co-culture; **(C)** Scratch wound healing assay measuring LLC migration capacity at 0, 12, and 24 hours(scale bar = 50 μm); **(D)** Transwell invasion assay results after 12 and 24 hours of co-culture (crystal violet staining, 100× magnification, scale bar = 50 μm); **P<0.01, ****P<0.0001, vs. Blank group; &&P<0.01, &&&P<0.001, &&&&P<0.0001, vs. CON group.

Thermally stressed Treg cells exhibited significantly attenuated tumor-promoting functions in our experimental system. Building upon previous findings, we employed a simplified 15-minute thermal stress protocol to evaluate how heat-treated Treg cells modulate Lewis Lung Carcinoma (LLC) immune evasion. High-resolution phase-contrast imaging revealed distinct morphological changes in LLC cells depending on Treg treatment: while control Treg co-cultures (CON) displayed characteristic tumorigenic phenotypes with dense, elongated cell arrangements, LLC cells co-cultured with heat-treated Tregs became sparse and rounded (**Figure 2A**). CCK-8 assays showed the pro-proliferative effect of Tregs was attenuated by heat treatment, with 55 °C-treated Tregs showing significantly weaker enhancement of LLC growth (**Figure 2B**, P < 0.0001). This functional impairment extended to metastatic behaviors, as scratch and Transwell assays confirmed heat-treated Tregs (45 °C/55 °C) reversed the >2-fold enhancement of migration and invasion seen with control Tregs (P < 0.0001), reducing these capacities by 40-60% (**Figures 2C, D**). Collectively, these results demonstrate that thermal stress ≥45 °C temperature-dependently disrupts Treg-mediated tumor support.

### Thermal ablation disrupts Treg-mediated immunosuppression to potentiate CD8+ T cell tumor killing

**Figure 3:**
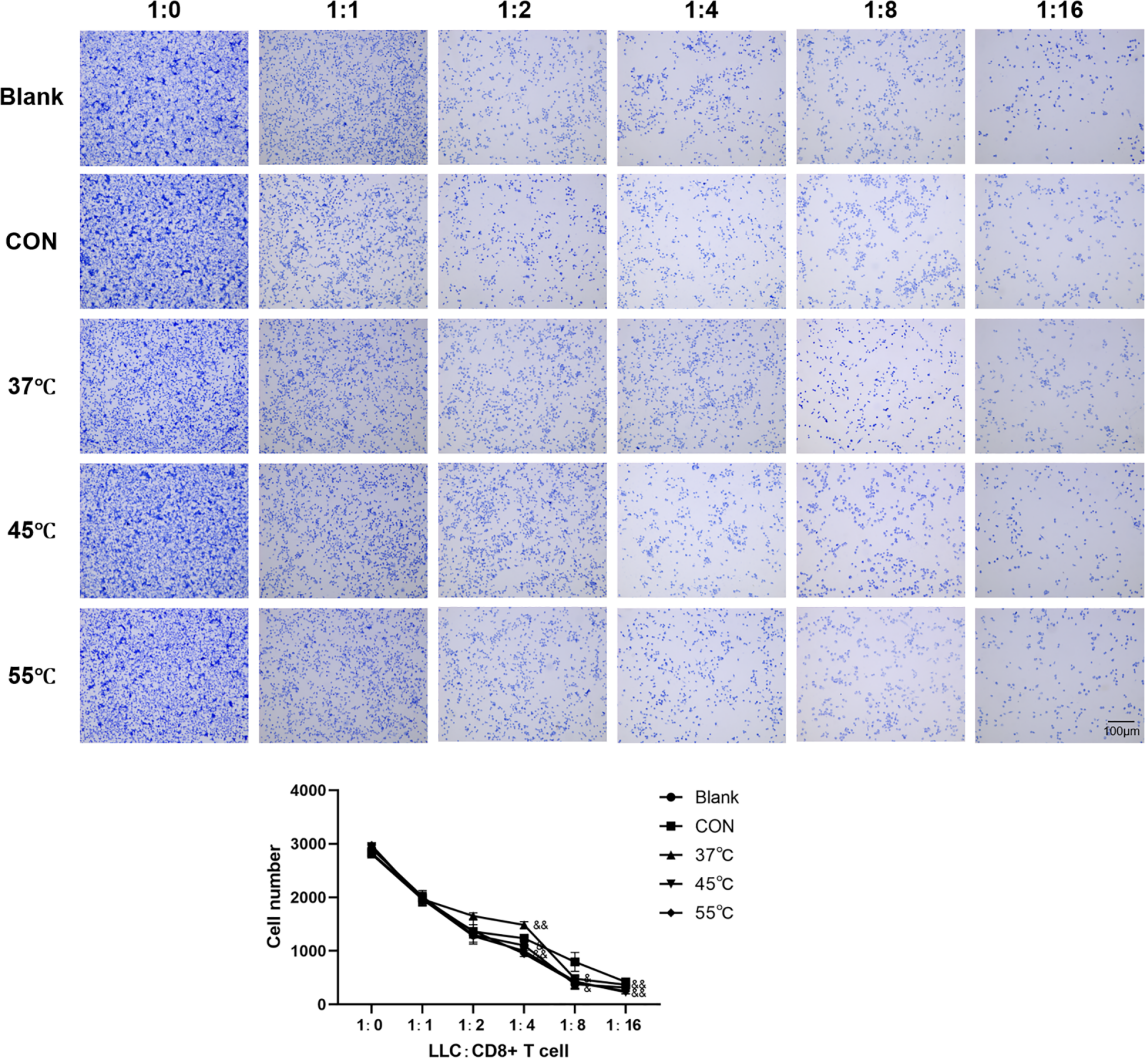
Thermal ablation disrupts Treg-mediated immunosuppression to potentiate CD8+ T cell tumor killing. Scale bar = 100μm; &P<0.05, &&P<0.01 vs. CON group.

In the lymphocyte cytotoxicity experiments, we first co-cultured five groups of Treg cells with LLC tumor cells for 24 hours. Highly purified CD8+ T cells were then isolated and co-cultured with the pre-treated LLC cells at varying effector-to-target ratios (1:0 to 1:16). Crystal violet staining and cell counting revealed findings: Increasing CD8+ T cell ratios led to progressive reduction in viable LLC cell numbers, confirming intact CD8+ T cell function. Control Tregs strongly protected LLC cells from CD8+ T cell killing (2.3-fold more surviving cells at 1:8 ratio vs. no-Treg control, p<0.001). The protective effect of Tregs for LLC cells from CD8+ T cell killing was significantly attenuated by 45 °C and 55 °C treatment (P<0.05, **Figure 3**).

### Thermal ablation disrupts Treg immunosuppression via the OX40L/NF-κB pathway

**Figure 4:**
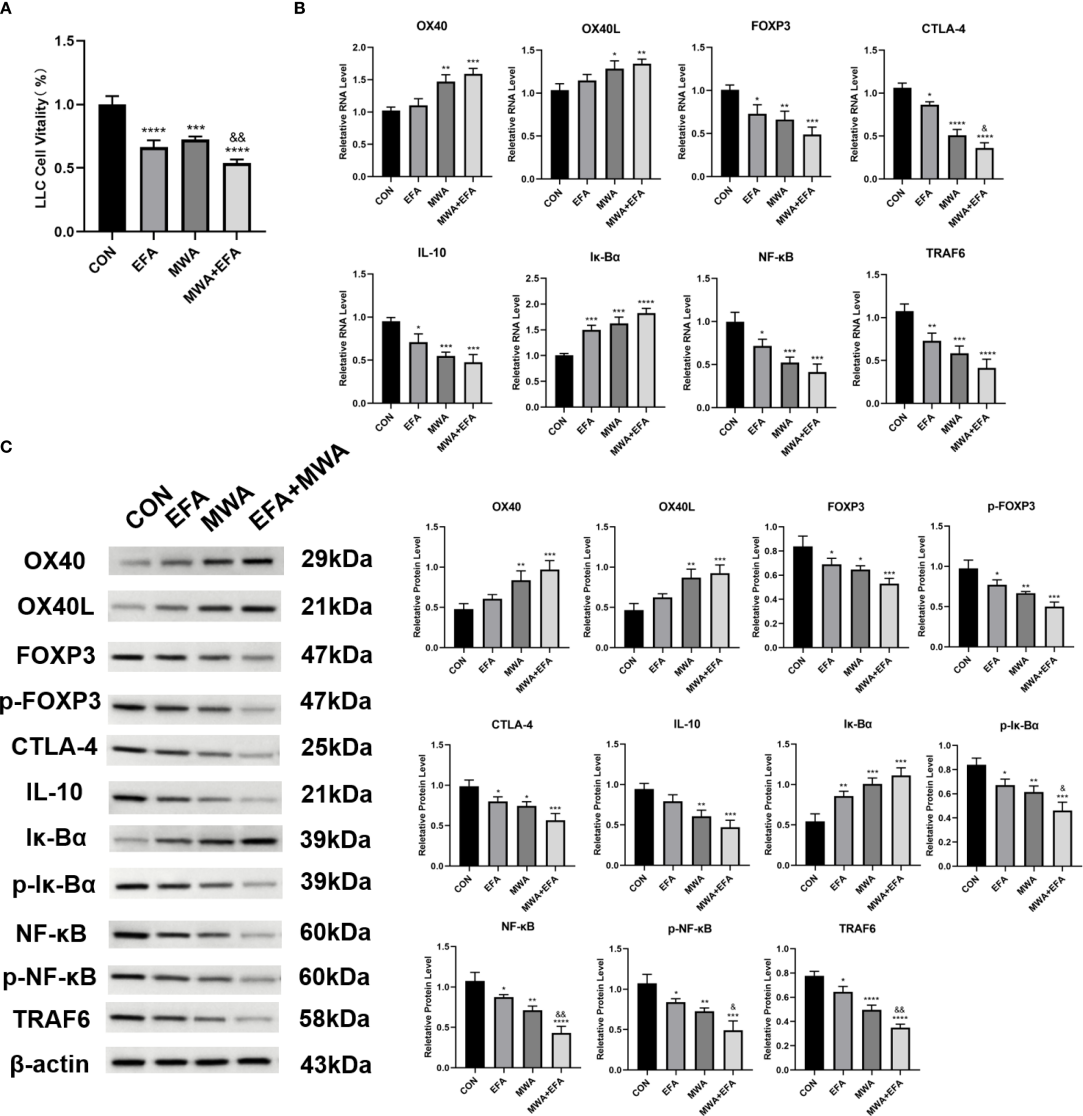
Thermal ablation and OX40 activation disrupt Treg immunosuppressive function. **(A)** CCK-8 assay of LLC proliferation after co-culture with treated Tregs; **(B)** RT-qPCR analysis of OX40/OX40L and immunosuppressive genes; **(C)** Western blot of NF-κB pathway and related proteins; *P<0.05, **P<0.01, ***P<0.001, ****P<0.0001, vs. CON group; &P<0.05, &&P<0.01, vs. MWA group.

Our mechanistic studies revealed that thermal ablation (55 °C, 15 min) and OX40 agonism (Efizonerimod alfa) collectively impaired Treg-mediated immunosuppression. CCK-8 assays demonstrated that both heat-stressed Tregs and OX40 agonist-treated Tregs significantly reduced LLC proliferation compared to the CON group (P < 0.001), with combined treatment showing enhanced suppression (P < 0.01 vs. MWA group) (**Figure 4A**). Molecular analyses identified coordinated regulation of key pathways: heat stress and OX40 activation upregulated IκBα expression (P < 0.01) while downregulating immunosuppressive markers FOXP3, CTLA-4 and IL-10 (P < 0.05) (**Figures 4B, C**). Notably, both interventions suppressed NF-κB pathway activation, as evidenced by reduced p-IκBα, NF-κB/p-NF-κB and TRAF6 levels (P< 0.05), with combined treatment producing the more pronounced inhibition (P < 0.05) (**Figures 4B, C**). The potent synergy between MWA and the specific OX40 agonist strongly suggests that the observed effects are mediated through the OX40L/TNFRSF4 axis.

### *In vivo* validation of thermal ablation effects on TNFRSF4+ Treg-mediated tumor immunity

**Figure 5:**
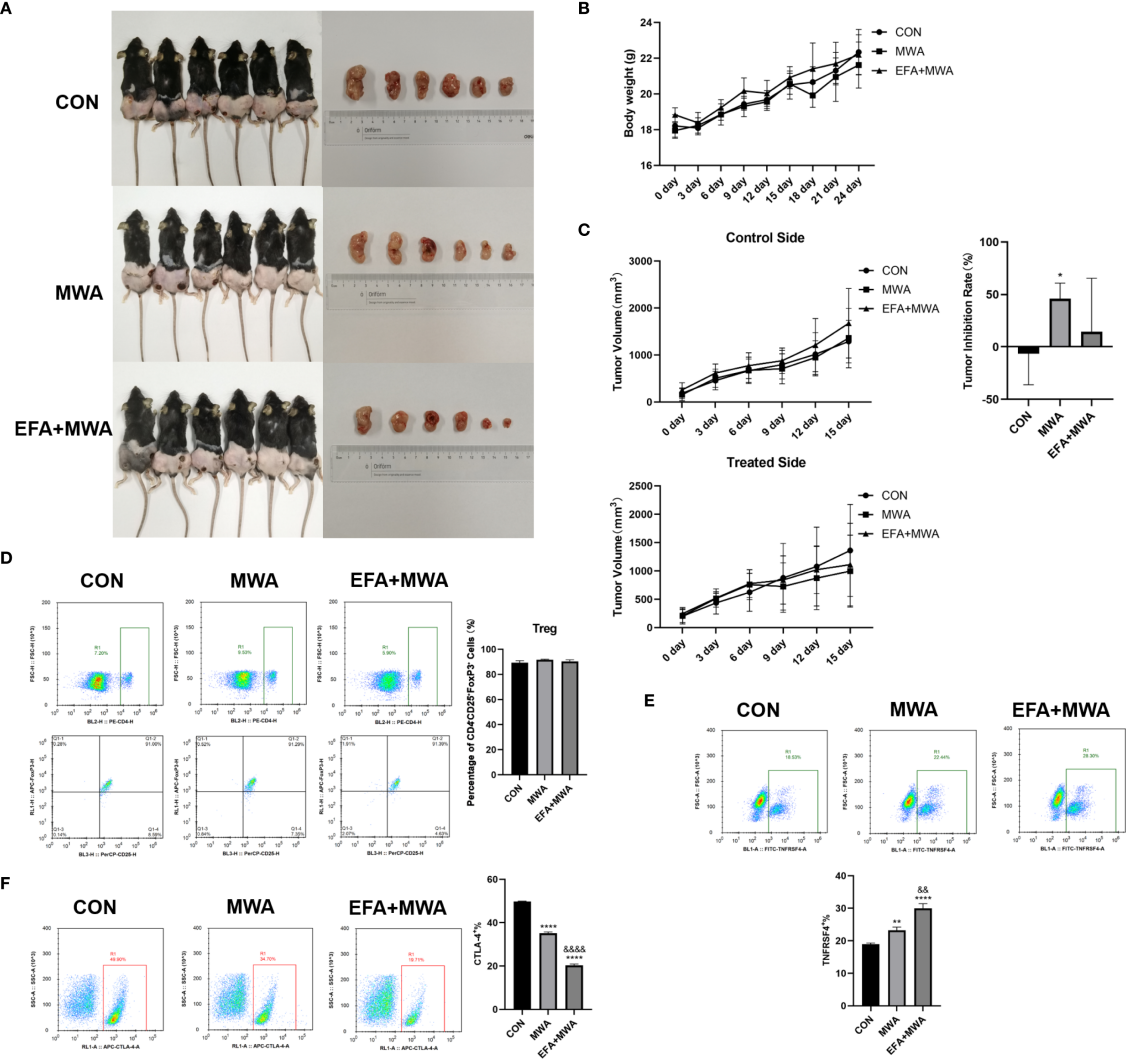
Thermal ablation effects on tumor growth and Treg phenotypes in vivo. **(A)** Representative images of tumor-bearing mice and excised tumors; **(B)** Body weight changes over time; **(C)** Tumor volume ratios (treated/contralateral) at endpoint; Flow cytometry of tumor-infiltrating Treg subsets: **(D)** CD4+CD25+FOXP3+, **(E)** TNFRSF4+, and **(F)** CTLA-4+ frequencies; *P<0.05, **P<0.01, ****P<0.0001, vs. CON group; &&P<0.01, &&&&P<0.0001, vs. MWA group.

To validate our mechanistic findings, we established LLC xenograft tumor models in mice and performed localized thermal ablation (55 °C, 15 min) with or without OX40 agonist treatment. **Figure 5A** shows representative pictures of the experimental mice as well as the tumors of the mice in each period. Longitudinal monitoring revealed that while all groups showed progressive tumor growth, thermal ablation significantly suppressed tumor volume in treated lesions compared to contralateral controls (P < 0.05, **Figure 5C**), without affecting mouse body weight (**Figure 5B**). Flow cytometry analysis of tumor-infiltrating Tregs demonstrated three key phenotypes: (1) Comparable total Treg frequencies across groups (**Figure 5D**); (2) Increased TNFRSF4+ subset in ablation-treated tumors (P < 0.01), further amplified by OX40 agonism (P < 0.01 vs MWA) (**Figure 5E**); and (3) Conversely, CTLA-4+ Tregs decreased post-ablation (P < 0.01) with additional reduction from OX40 activation (P < 0.01) (**Figure 5F**).

**Figure 6:**
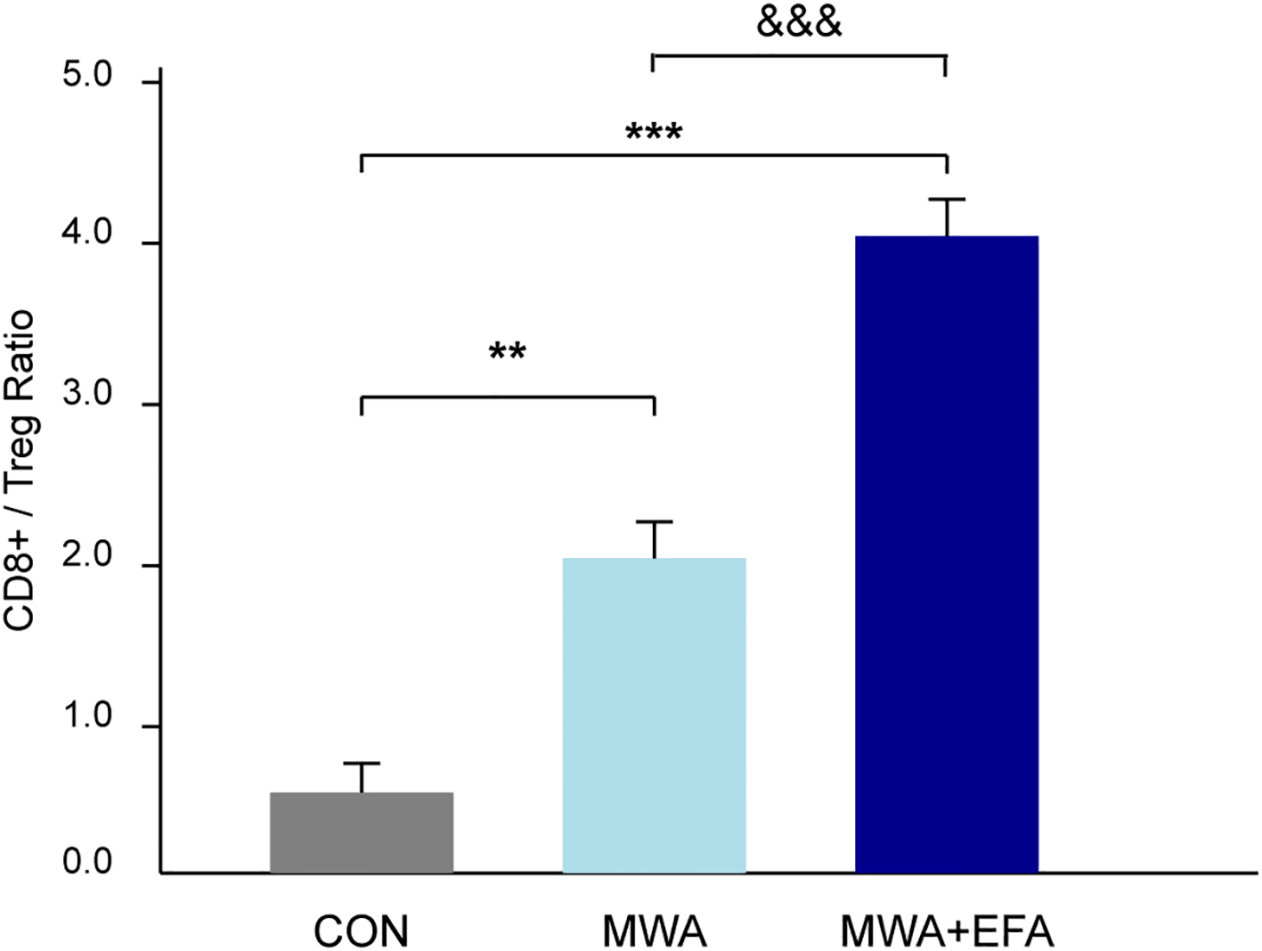
MWA combined with OX40 agonism shifts the tumor immune balance towards a cytotoxic profile. The ratio of intratumoral CD8+ T cells to FOXP3+ Tregs was quantified by flow cytometry from dissociated tumors of each experimental group. The combination of MWA and EFA leads to a significantly higher ratio, indicating a shift from an immunosuppressive to a pro-inflammatory microenvironment. Data are mean ± SD. **P<0.01, ***P<0.001, vs. CON group; &&&P<0.001 vs. MWA group.

To more broadly assess the impact of MWA on the tumor immune microenvironment, we next quantified the balance between pro-tumor immunosuppressive cells and anti-tumor effector cells. We calculated the ratio of infiltrating cytotoxic CD8+ T cells to FOXP3+ Tregs using our flow cytometry data. As shown in **Figure 6**, the CD8+/Treg ratio in control tumors was low, indicating an immunosuppressive environment. MWA alone dramatically increased this ratio (P < 0.01), and the combination of MWA with the OX40 agonist (EFA) led to a profound and highly significant further increase (P < 0.001 vs CON; P < 0.001 vs MWA). This result provides strong quantitative evidence suggesting a substantive shift of the TME from a suppressive to a cytotoxic-dominant state following our combination therapy.

**Figure 7:**
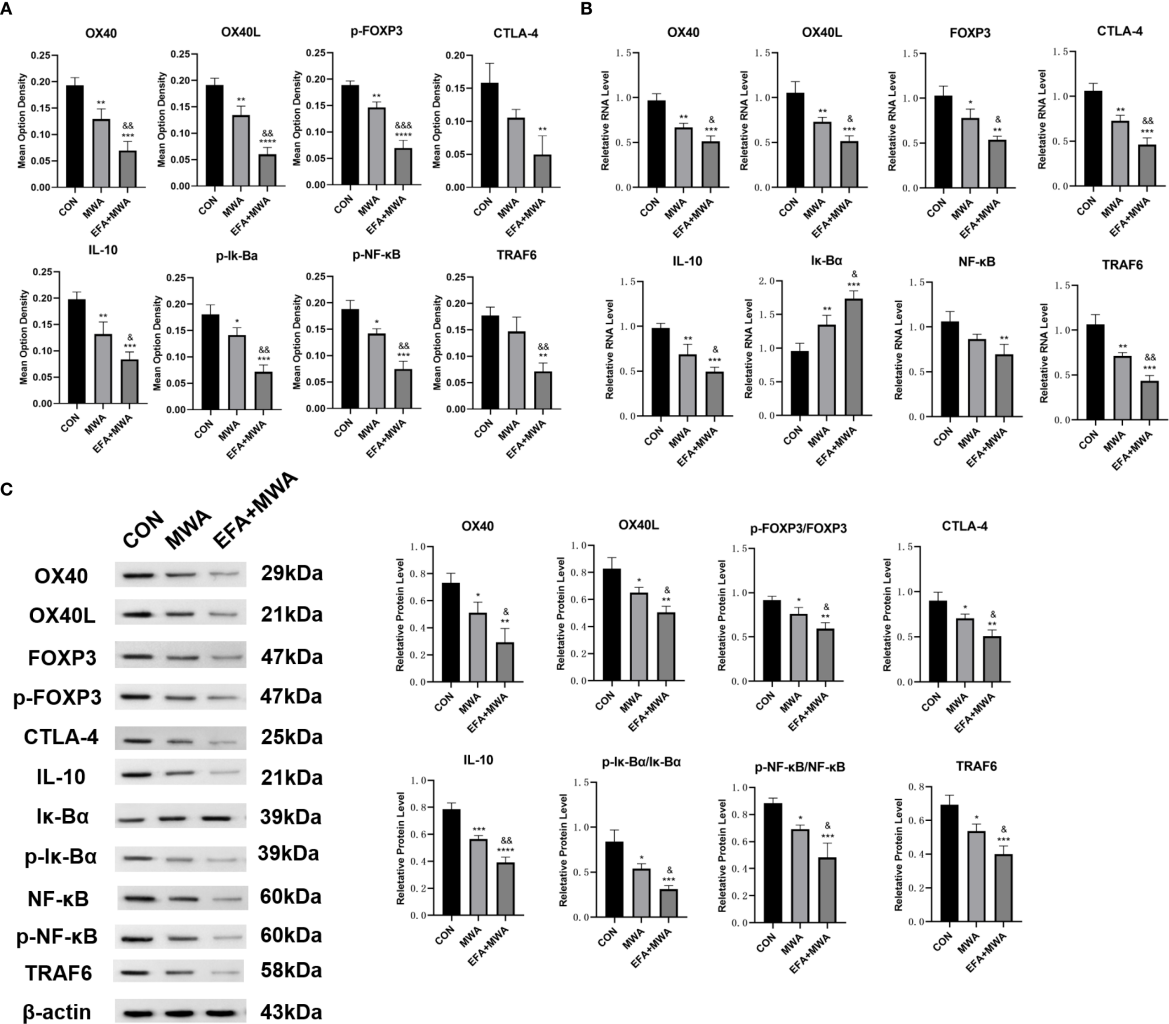
Molecular signatures in microwave ablated tumors. **(A)** Immunohistochemistry of immunosuppressive genes in tumors; **(B)** RT-qPCR analysis of immunosuppressive genes; **(C)** Western blots of immunosuppressive proteins; *P<0.05, **P<0.01, ***P<0.001, vs. CON group; &P<0.05, &&P<0.01, vs. MWA group.

To investigate the underlying molecular mechanisms for these cellular changes, we analyzed tumor tissues. Representative immunohistochemical staining demonstrated reduced expression of the Treg master regulator FOXP3 in the MWA and MWA+EFA groups, consistent with Treg destabilization (**Figure 7A**). Furthermore, molecular profiling (**Figures 7B, C**) confirmed coordinated downregulation of immunosuppressive factors (IL-10) and key NF-κB pathway components (phospho-IκBα, phospho-NF-κB, TRAF6) in MWA groups (P < 0.05), with the strongest suppression observed in the combination therapy group (P < 0.05). These *in vivo* results substantiate that thermal ablation disrupts Treg function via inhibition of the OX40L/NF-κB axis.

## Discussion

Our study demonstrates that microwave ablation (MWA) disrupts TNFRSF4+ Treg-mediated immunosuppression, potentially through OX40L/NF-κB pathway inhibition. *In vitro*, thermal stress attenuated Tregs’ pro-tumor functions (proliferation/migration/invasion support) while enhancing CD8+ T cell cytotoxicity. Mechanistically, heat-treated Tregs exhibited upregulated OX40L but downregulated CTLA-4/IL-10 and NF-κB activity, effects amplified by OX40 agonism. *In vivo*, localized ablation selectively suppressed tumor growth without systemic toxicity, increasing TNFRSF4+ while decreasing CTLA-4+ Treg subsets. Critically, our quantitative analysis reveals that this combination therapy appears to significantly reshape the tumor immune microenvironment by substantially boosting the CD8+/Treg ratio, providing strong evidence of a shift from an immunosuppressive to an anti-tumor state.

The growing adoption of thermal ablation techniques, particularly MWA, as minimally invasive alternatives for treating solid tumors has unveiled their dual role in both direct tumor destruction and immunomodulation (15, 16). While MWA’s clinical efficacy in lung cancer management, especially for inoperable patients, is well-established through its ability to induce coagulative necrosis and achieve superior local tumor control compared to radiofrequency ablation (17–19), emerging evidence highlights its profound capacity to reshape the tumor immune microenvironment (20, 21). Our new data showing the marked increase in the CD8+/Treg ratio provides strong quantitative evidence for this potential reprogramming toward an immune-active state. This ratio is a widely accepted biomarker of immune competence within the TME, where a higher value is strongly associated with favorable patient outcomes and better responses to immunotherapy across numerous cancers (22–24). Our results therefore strongly suggest the therapeutic potential of MWA to convert an immunosuppressive ‘cold’ tumor into an inflamed ‘hot’ tumor, rich in cytotoxic effector cells.

The immunogenic effects of thermal ablation have been attributed to its ability to release tumor antigens and danger signals, effectively creating an *in-situ* vaccine that primes antitumor immunity (21, 25). This immunogenic cell death, a cornerstone of the post-ablation immune response, is increasingly being harnessed by novel theranostic nanoplatforms that combine imaging with phototherapy to maximize immune stimulation (26). This phenomenon has been observed across multiple cancer types, including lung (27), breast (28), and hepatocellular carcinomas (29). However, the specific mechanisms by which hyperthermia modulates immune cell subsets remain poorly defined. Our study advances this understanding by demonstrating that MWA selectively disrupts the function of TNFRSF4+ Tregs. The stable total Treg frequency (**Figure 5D**) alongside a significant decrease in the highly suppressive CTLA-4+ subset (**Figure 5F**) and a concurrent increase in the TNFRSF4+ subset (**Figure 5E**) suggests a complex phenotypic reprogramming. MWA appears to shift the Treg population from a stable, highly suppressive state (CTLA-4 high) towards a more plastic phenotype (TNFRSF4 high) that is amenable to OX40 agonist therapy. This reprogramming, rather than simple depletion, is a key finding of our study.

Tregs are pivotal in maintaining immune tolerance and fostering tumor immune evasion. Our data reveal that thermal stress attenuates Tregs’ pro-tumor functions, including their capacity to support tumor proliferation, migration, and invasion. This is consistent with prior studies showing that OX40 signaling can inhibit Treg immunosuppressive activity by downregulating FOXP3 and CTLA-4 expression, thereby reducing IL-10 production and abrogating Treg-mediated suppression of effector T cells (4, 30). Notably, we observed that heat-treated Tregs exhibit diminished NF-κB activity, a pathway essential for Treg survival and function (31). The downregulation of NF-κB likely contributes to the reduced stability of Tregs in the tumor microenvironment (TME) post-ablation, further shifting the balance toward immune activation.

Some studies only assessed the immune status at a specific time point after ablation, limiting the ability to track the dynamic effect of MWA on the immune response. Animal experiments involving lung cancer are scarce. The complexity of the *in vivo* immune network requires a more nuanced approach, for which animal models provide an indispensable tool. The Lewis lung cancer (LLC) mouse model serves as an archetype for this purpose. It has an important role in biological and translational studies of lung cancer with broad application in the field of immunology (32). *In vivo*, localized MWA selectively suppressed tumor growth without systemic toxicity, coinciding with a reduction in CTLA-4+ Treg subsets and an increase in TNFRSF4+ Tregs. This phenotypic shift suggests that thermal ablation may preferentially target highly immunosuppressive Treg populations while sparing or even enhancing subsets amenable to OX40-mediated immune stimulation. The selective depletion of immunosuppressive Tregs aligns with preclinical studies demonstrating that OX40 agonists can deplete intratumoral Tregs via FcγR-mediated mechanisms, thereby enhancing anti-tumor immunity (33).

OX40L (CD134L), a member of the tumor necrosis factor superfamily, is mainly expressed on the surface of antigen-presenting cells (APCs) and some immune cells. When combined with OX40, it can activate the OX40 signaling pathway, thereby enhancing the survival, proliferation and cytotoxicity of T cells. The OX40/OX40L axis plays a dual role in immune regulation, acting as both a co-stimulatory signal for effector T cells and a modulator of Treg function. Our findings indicate that thermal ablation upregulates OX40L expression on Tregs while simultaneously inhibiting their immunosuppressive capacity. This paradoxical effect may reflect a compensatory response to thermal stress, wherein Tregs transiently upregulate OX40L as part of a pro-inflammatory shift. Importantly, OX40 agonism further amplified the ablation-induced suppression of Treg function, suggesting that combining thermal ablation with OX40-targeted therapies could maximize immune activation.

Mechanistically, the downregulation of NF-κB activity in heat-treated Tregs likely disrupts their survival and functional stability, as NF-κB is a critical downstream effector of OX40 signaling (34). The synergy between ablation and OX40 agonism may thus arise from complementary mechanisms: ablation disrupts Treg immunosuppression by inhibiting NF-κB, while OX40 agonists enhance effector T cell responses by promoting proliferation, cytokine production, and memory formation (35). This dual approach aligns with recent clinical observations that OX40 agonists, though limited as monotherapy, may exhibit greater efficacy when combined with modalities that alter the TME, such as checkpoint inhibitors or radiation (36, 37).

Clinically, the synergy between MWA and immune checkpoint inhibitors (e.g., anti-PD-1/PD-L1) has shown promise (38, 39), yet the underlying immunological basis has been unclear. Our work helps provide mechanistic clarity by suggesting that the OX40L/TNFRSF4 axis as a thermally sensitive regulatory node in Tregs and demonstrating its potential to favorably alter the TME. Our quantitative data on the CD8+/Treg ratio provides a strong foundation and a clear biomarker for the rationale of this combination therapy. A limitation of our study is the absence of genetic knockout experiments to definitively confirm the dependency on OX40L. However, the strong synergistic effects observed with the pharmacological OX40 agonist provide compelling indirect evidence for this pathway’s involvement and offer a promising avenue for future investigation. Furthermore, to assess the broader applicability of our findings, we confirmed the synergistic anti-tumor effects of MWA and OX40 agonism in additional preclinical models of B16-F10 melanoma and 4T1 breast cancer (**Supplementary Figure 1**), suggesting the potential for broader translational relevance.

Despite the promising preclinical results, the translational potential of OX40-targeted therapies has been hampered by variable patient responses, likely due to heterogeneous OX40/OX40L expression across tumors (4). Our data suggest that thermal ablation could serve as a priming strategy to sensitize tumors to OX40 agonism by reducing Treg-mediated suppression and creating a more immunogenic TME. Future studies should explore whether ablation-induced OX40L upregulation correlates with enhanced clinical responses to OX40 agonists, particularly in tumors with low baseline OX40 expression.

Moreover, the timing and sequencing of ablation with OX40-targeted therapies warrant investigation. Preclinical models indicate that sequential administration, where ablation precedes OX40 agonism, may optimize immune activation by first disrupting immunosuppressive networks before stimulating effector responses (40). This approach could mitigate the exhaustion observed with concurrent high-dose OX40 agonist therapy (36).

Our study builds upon these observations by demonstrating that MWA directly modulates Treg function through the OX40L/TNFRSF4 axis. The thermal stress induced by MWA triggers OX40L upregulation in Tregs, leading to the disruption of their immunosuppressive capacity. This effect is mechanistically distinct from but potentially complementary to TGF-β pathway inhibition (41), as both strategies target Treg activity in the tumor microenvironment. Together, these findings suggest that MWA may prime the immune system for enhanced responses to checkpoint inhibitors by simultaneously activating effector T cells and inhibiting Treg-mediated suppression. Future studies should explore optimal combinations of MWA with targeted immunomodulators to maximize therapeutic outcomes.

## Conclusion

**Figure 8:**
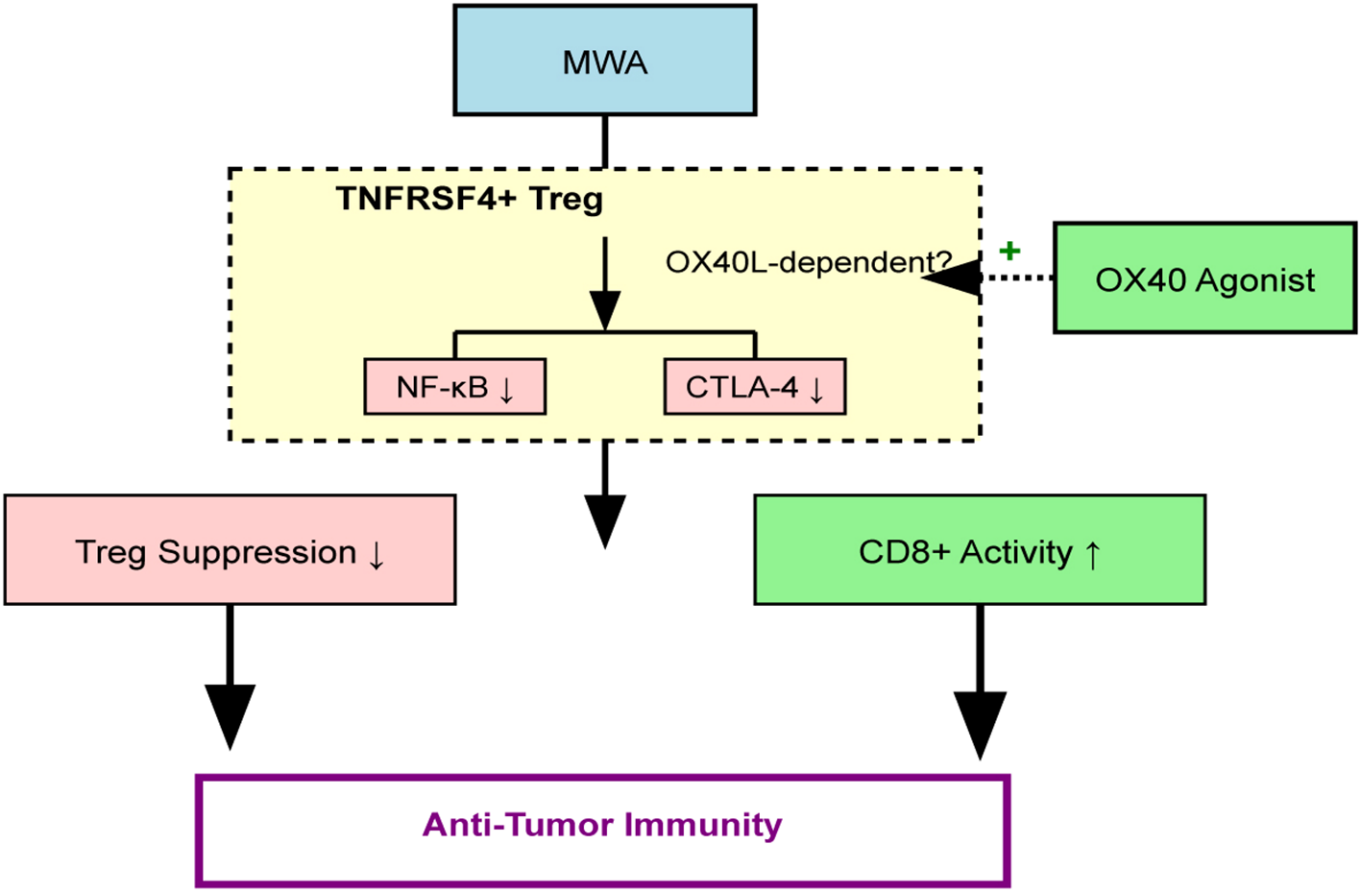
Schematic of the proposed mechanism. MWA induces thermal stress, which promotes OX40L-dependent inhibition of the NF-κB pathway in TNFRSF4+ Tregs. This impairs Treg immunosuppressive function (e.g., reduced CTLA-4, IL-10) and enhances CD8+ T cell cytotoxicity. An OX40 agonist (EFA) potentiates this effect, leading to a profound shift in the TME balance (increased CD8+/Treg ratio) and enhanced tumor control.

MWA-like heat stress irreversibly impairs TNFRSF4+ Treg function, likely via OX40L/NF-κB signaling, mitigating their protumor effects. Combining MWA with OX40 agonists enhances antitumor immunity, partially by targeting Treg suppression and promoting CD8+ T cell activation. This is quantitatively demonstrated by a significant improvement in the intratumoral CD8+/Treg ratio, suggesting a significant reprogramming of the TME from a suppressive to an effector-dominant state. By destabilizing Treg immunosuppression and enhancing OX40 costimulation, this strategy may overcome resistance mechanisms that limit current immunotherapies. Future work should validate these findings in clinical trials, with a focus on biomarker-driven approaches, such as the CD8+/Treg ratio, to harness the full potential of OX40/OX40L modulation. The overarching mechanism is summarized in **Figure 8**.

## Data Availability

The original contributions presented in the study are included in the article/**Supplementary Material**. Further inquiries can be directed to the corresponding authors.

## Supplementary material

Supplementary Figure 1 MWA and OX40 agonist combination therapy shows efficacy in other tumor models. Tumor growth curves for mice bearing **(A)** B16-F10 melanoma or **(B)** 4T1 breast cancer tumors, treated with control, MWA, or MWA+EFA. The combination therapy significantly suppresses tumor growth in both models. *P<0.05 vs MWA group.

Supplementary Figure 2 Representative flow cytometry plots for CTLA-4+ Treg cell frequency. The plots correspond to the quantification shown in **Figure 1D**, demonstrating the gating strategy and the reduction in CTLA-4 expression on Tregs after 6 hours of MWA-mimetic thermal stress at different temperatures and durations.
