# Supplementary material for: Microwave ablation triggers OX40L-mediated disruption of TNFRSF4+ Treg immunosuppressive activity
Source: Front Immunol. 2025 Oct 23;16:1637317. doi: 10.3389/fimmu.2025.1637317 (PMC12588988; doi:10.3389/fimmu.2025.1637317)
Supplement: Supplementary Figure 1 — MWA and OX40 agonist combination therapy shows efficacy in other tumor models. Tumor growth curves for mice bearing (A) B16-F10 melanoma or (B) 4T1 breast cancer tumors, treated with control, MWA, or MWA+EFA. The combination therapy significantly suppresses tumor growth in both models. *P<0.05 vs MWA group. [file Table1.docx]

Supplementary Materials

Supplementary Table 1. Primers sequence

| **Gene** | **Primer** | |
| --- | --- | --- |
| OX40/TNFRSF4 | F | TGCCTAACACTCCCAAACCT |
|  | R | AAGTGTGCGTCTGTGTGTTC |
| OX40L/TNFSF4 | F | GGTCTCTGGGATCAAGGGAG |
|  | R | AGAGAGTTGCAGGCAGACAT |
| FOXP3 | F | CTGCTGGCAAATGGAGTCTG |
|  | R | CTGCACCACTTCTCTCTGGA |
| IL-10 | F | TGTAGCAGTCTTGACGCAGA |
|  | R | CCGTGGATAGAGGCTAGGTG |
| NF-κB | F | TTGGGAGAAGGCTGGAGAAG |
|  | R | TGAACACAGGCTCATACGGT |
| TRAF6 | F | ACTAGTGCGCTGTGAAGTCT |
|  | R | GCATCAGTACTTCGTGGCTG |
| CTLA-4 | F | TTGACACGGGACTGTACCTC |
|  | R | AAAGTATGGCGGTGGGTACA |
| Iκ-Bα | F | GTAGCAGTCTTGACGCAGACCT |
|  | R | ACATCAGCACCCAAAGTCACCAA |
| GAPDH | F | CTCCTGAGCGCAAGTACTCT |
|  | R | TACTCCTGCTTGCTGATCCAC |

Supplementary Table 2. Information of antibodies

| **Name** | **Producer** | **Cat. #** | **Dilution Ratio** | **Purpose** |
| --- | --- | --- | --- | --- |
| TNFRSF4/CD134 Antibody | Affinity | DF7751 | 1：1000 | Western Blot |
| TNFSF4 Antibody | Affinity | DF7816 | 1：2000 |  |
| TRAF6 Antibody | Affinity | AF5376 | 1：2000 |  |
| IKB alpha Antibody | Affinity | AF5002 | 1：1000 |  |
| Phospho-IKB alpha (Ser32/Ser36) Antibody | Affinity | AF2002 | 1：1000 |  |
| NF-kB p65 Antibody | Affinity | AF5006 | 1：1000 |  |
| Phospho-NF-kB p65 (Ser536) Antibody | Affinity | AF2006 | 1：1000 |  |
| IL10 Antibody | Affinity | DF6894 | 1：1000 |  |
| Foxp3 Antibody | Affinity | AF6544 | 1：1000 |  |
| Phospho-Foxp3 (Ser19) Antibody | Affinity | AF3544 | 1：1000 |  |
| CTLA4 Antibody | Affinity | DF6793 | 1：1000 |  |
| HRP-Goat Anti-Rabbit IgG | Servicebio | C030212 | 1：5000 | Secondary antibody |
| TNFRSF4/CD134 Antibody | Affinity | DF7751 | 1：50 | IHC |
| TNFSF4 Antibody | Affinity | DF7816 | 1：50 |  |
| TRAF6 Antibody | Affinity | AF5376 | 1：50 |  |
| IKB alpha Antibody | Affinity | AF5002 | 1：50 |  |
| Phospho-IKB alpha (Ser32/Ser36) Antibody | Affinity | AF2002 | 1：50 |  |
| NF-kB p65 Antibody | Affinity | AF5006 | 1：50 |  |
| Phospho-NF-kB p65 (Ser536) Antibody | Affinity | AF2006 | 1：50 |  |
| IL10 Antibody | Affinity | DF6894 | 1：50 |  |
| Foxp3 Antibody | Affinity | AF6544 | 1：50 |  |
| Phospho-Foxp3 (Ser19) Antibody | Affinity | AF3544 | 1：50 |  |
| CTLA4 Antibody | Affinity | DF6793 | 1：50 |  |
| HRP PcAb Goat Anti-Rabbit IgG（H+L） | Servicebio | C030212 | 1：200 | Secondary antibody |
| HRP PcAb Goat Anti-Mouse IgG（H+L） | Servicebio | C030205 | 1：200 | Secondary antibody |


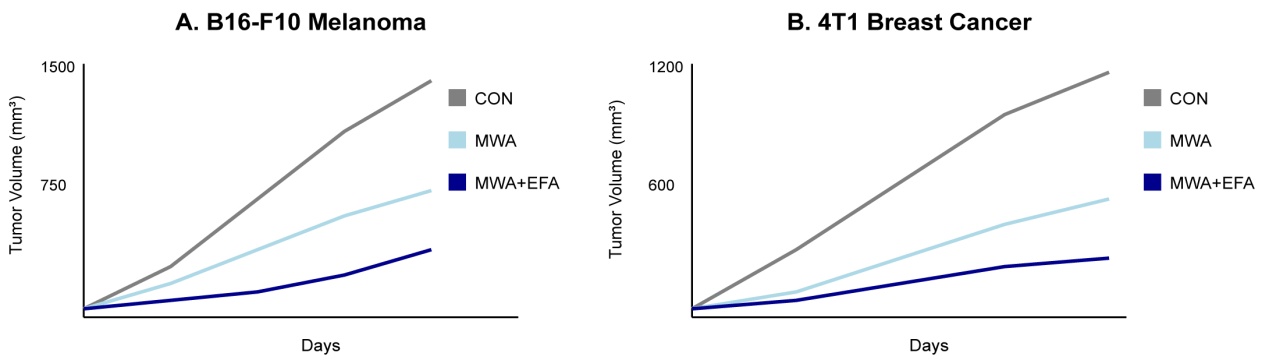


**Supplementary Figure 1. MWA and OX40 agonist combination therapy shows efficacy in other tumor models.**Tumor growth curves for mice bearing (A) B16-F10 melanoma or (B) 4T1 breast cancer tumors, treated with control, MWA, or MWA+EFA. The combination therapy significantly suppresses tumor growth in both models. *P<0.05 vs MWA group.


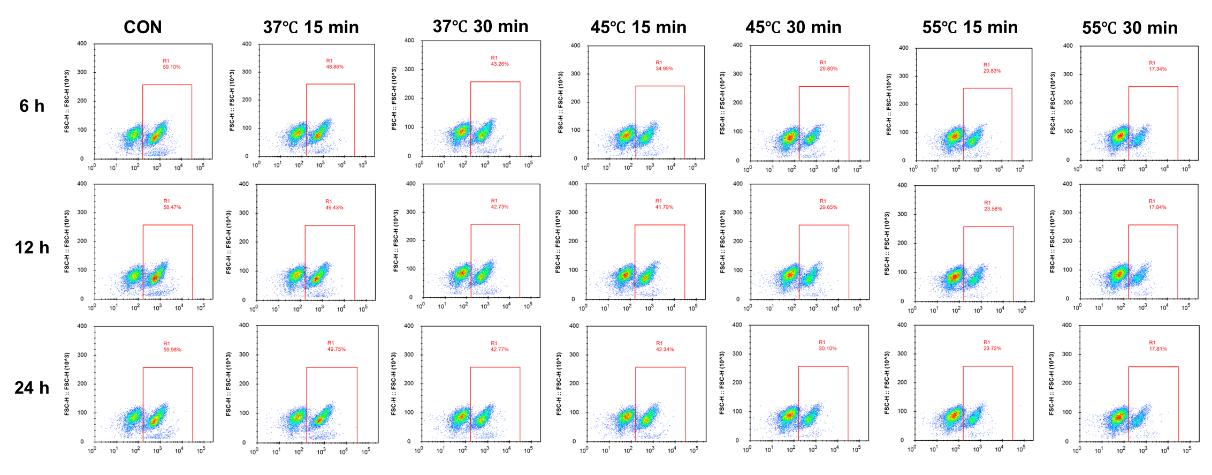


**Supplementary Figure 2.** **Representative pictures of CTLA-4^+^ Treg cell frequency (Figure 1D).**
